# The Protective Effect of rhBNP on Postresuscitation Myocardial Dysfunction in a Rat Cardiac Arrest Model

**DOI:** 10.1155/2020/6969053

**Published:** 2020-02-17

**Authors:** Min Yang, Tianfeng Hua, Zhengfei Yang, Limin Chen, Yangyang Zou, Xiaohui Huang, Jun Li

**Affiliations:** ^1^Department of Basic and Clinical Pharmacology, School of Pharmacy, Anhui Medical University, Hefei, China; ^2^Department of Intensive Care Unit, The Second Affiliated Hospital of Anhui Medical University, Hefei, China; ^3^Institute of Cardiopulmonary Cerebral Resuscitation, Sun Yat-sen University, Guangzhou, China

## Abstract

**Purpose:**

We investigated the protective effects and the underlying mechanisms through which recombinant human brain natriuretic peptide (rhBNP) acts on postresuscitation myocardial dysfunction (PRMD) in the cardiac arrest (CA) model.

**Methods:**

Ventricular fibrillation was induced and untreated for 6 min. And the time of cardiopulmonary resuscitation was 8 min, after which defibrillation was attempted in this rat model. 24 Sprague Dawley rats (450–550g) were randomized into cardiopulmonary resuscitation (CPR) + rhBNP and CPR + placebo groups after restoration of spontaneous circulation (ROSC). rhBNP was infused at PR 30 min (loading dose: 1.5 *µ*g/kg, 3 min; maintenance dose: 0.01 *µ*g/kg, 3 min; maintenance dose: 0.01 *α* (TNF-*α* (TNF-*α* (TNF-*κ*B (NF-*κ*B (NF-

**Results:**

The administration of rhBNP attenuated the severity of PRMD and myocardial tissue injuries, with improvement of MAP (mean arterial blood pressure), ETCO_2_ (end-tidal CO_2_), serum level of NT-proBNP, EF, CO, and MPI values. The serum levels and protein expression levels in myocardial tissue of IL-6 and TNF-*α* (TNF-*κ*B (NF-

**Conclusion:**

Our research demonstrated that the administration of rhBNP attenuated the severity of PRMD and myocardial tissue injuries and increased the 24 h survival rate in this CA model. rhBNP administration also reduced the serum and myocardial tissue levels of IL-6 and TNF-*α* after ROSC, likely due to the suppression of the TLR4/NF-*κ*B signaling pathway and the regulation of inflammatory mediator secretion.*α* (TNF-*κ*B (NF-

## 1. Introduction

Cardiopulmonary resuscitation (CPR) improves the restoration of spontaneous circulation (ROSC) rate in cardiac arrest (CA) patients. The survival rate following CA remains unsatisfactory, and the survival-to-discharge ratios following out-of-hospital and in-hospital adult CA episodes are 7.9% and 21%, respectively, in the USA [1]. The severity of postresuscitation syndrome (PRS) is closely related to both the survival rates and the long-term outcomes of CA patients, with more than 70% of patients dying within 72 h after ROSC [2, 3]. Because CA and following CPR result in systemic ischemia/reperfusion (I/R) injuries, the primary manifestations of PRS include postresuscitation (PR) brain injuries, postresuscitation myocardial dysfunction (PRMD), the systemic I/R response, and the persistent precipitating pathology [2].

PRMD is characterized by systolic and diastolic dysfunction and is closely related to early death after ROSC, especially during the first 24 h [4, 5]. The mechanism underlying PRMD is associated with myocardial stunning and myocardial tissue damage, as reflected by the significant increases in myocardial injury biomarkers that have been observed [4, 6]. Our previous study demonstrated that N-terminal probrain natriuretic peptide (NT-proBNP) and troponin I are both significantly correlated with the severity of PRMD and the prognosis following CA [6]. Recombinant human brain natriuretic peptide (rhBNP), a man-made peptide developed through gene engineering, has been widely used to treat acute heart failure induced by various causes. Research has suggested that rhBNP administration was able to significantly lower the serum concentrations of cardiac troponin T and NT-proBNP and improve myocardial function in patients with acute myocardial infarctions [7–9]. However, the protective effects of rhBNP on PRMD remain unclear. In the present study, we hypothesized that rhBNP could alleviate the severity of PRMD and improve the 24 h survival rate after CA. We further investigated the underlying protective mechanism of rhBNP on PRMD in a rat CPR model.

I/R injury involves multiple pathophysiological processes, including oxidative stress and calcium overload [10, 11]. Inflammatory mechanisms have been shown to play indispensable roles in the development of I/R injury [12]. The activation of nuclear transcription factor kappa B (NF-*κ*B) subunit p65 (p65) caused inflammatory cells to increase their expression and synthesis of proinflammatory factors [13, 14]. Among the many Toll-like receptor (TLR) signaling pathways, the activation of TLR4 is thought to be detrimental due to its effect on inflammatory signaling pathways [15]. During I/R injury, the TLR4/NF-*κ*B signaling pathway becomes activated, resulting in the secretion of many inflammatory cytokines that amplify the inflammatory response. Gui et al. demonstrated that the downregulation of the TLR4/NF-*κ*B pathway reduced the myocardial infarct size and improved cardiac function [16]. Recent research has demonstrated that rhBNP inhibits the expression of inflammatory factors (interleukin-6 (IL-6), IL-1*β*, and tumor necrosis factor-*α* (TNF-*α*)) and plays an anti-inflammation role during heart failure, systemic inflammatory response syndrome, myocardial inflammatory reaction, and lung injury by regulating the NF-*κ*B pathway [17–19]. Therefore, we hypothesize that rhBNP may regulate the TLR4/NF-*κ*B signaling pathway to alleviate the severity of PRMD.

## 2. Materials and Methods

### 2.1. Experimental Animals

We used twenty-four healthy, male, Sprague Dawley rats, weighing 450–550 g, which were supplied by a single source breeder (Experimental Animal Center of Sun Yat-sen University, Guangzhou, China). This study was approved by the Institutional Animal Care and Use Committee of Anhui Medical University and the Institute of Cardiopulmonary Cerebral Resuscitation of Sun Yat-sen University (R1709). All animals received humane care, in compliance with the Principles of Laboratory Animal Care formulated by the National Society for Medical Research and the Guide for the Care and Use of Laboratory Animals published by the National Institutes of Health [20, 21].

### 2.2. Experimental Procedures

All animals were intraperitoneally injected with pentobarbital (45 mg/kg) (Sigma-Aldrich, St. Louis, MO) for anesthesia, and additional doses (10 mg/kg) were administrated at 1 h intervals, except for 30 min prior to the induction of CA. The trachea was orally intubated with a 14 G cannula (Abbocath-T, North Chicago, IL). One polyethylene (PE-50) catheter (Abbocath-T, North Chicago, IL) was advanced into the descending aorta from the left femoral artery to measure arterial pressure (AP) and to collect blood samples for further analysis. Another PE-50 catheter was advanced into the right atrium through the left external jugular vein to measure right atrial pressure (RAP) and for drug infusion. All catheters were intermittently flushed with saline containing 2.5 IU/mL of crystalline bovine heparin. AP and RAP were measured by high-sensitivity transducers (Abbott Critical Care Systems, North Chicago, IL) and a MINDRAY monitoring system (BeneView T5, Shenzhen, China). End-tidal CO_2_ (ETCO_2_) was continuously monitored by the Capstar-100 CO_2_ analyzer (IITC Life Science, Ardmore, PA). A conventional lead II electrocardiogram (EKG) was also continuously monitored. A 3 F PE catheter (model C-PMS-301J; Cook Critical Care, Bloomington, Indiana) was advanced into the right atrium through the right external jugular vein, the position of which was confirmed by echocardiogram (ACUSON sc2000; Siemens, Munich, Germany). Then, a precurved guide wire was advanced into the right ventricle through the 3 F PE catheter to induce ventricular fibrillation (VF). The animals continued to spontaneously breathe normal air during preparation. During the experiment, the rectal temperatures of all rats were measured by the MINDRAY monitoring system, and the temperature were maintained at 37.0°C ± 0.2°C with a heating lamp.

Thirty minutes before VF, the animals were randomized into two groups by the sealed envelope method. (1) CPR + rhBNP group (*n* = 12); epinephrine (20 *µ*g/kg) was administered as a bolus injection into the right atrium after precordial compression (PC) was performed for 4 min. Thirty minutes after ROSC, rhBNP (Nuodikang Biological Pharmaceutical Company Ltd., Chengdu, China) was administered by continuous intravenous infusion (loading dose: 1.5 *µ*g/kg over 3–5 min; maintenance dose: 0.01 *µ*g/kg/min for 6 h). (2) CPR + placebo group (*n* = 12): epinephrine (20 *µ*g/kg) was administered as described for the previous group. A volume of saline equal to the volume of rhBNP used in the previous group was infused 30 min after ROSC. The resuscitated rats in each group were then randomly divided into two subgroups: ROSC 6 h (*n* = 6) and ROSC 24 h (*n* = 6). Twenty minutes before inducing VF, baseline (BL) measurements and arterial blood gas measurements were obtained. Fifteen minutes before inducing VF, mechanical ventilation was established at a tidal volume of 0.60 mL/100 g of body weight and a frequency of 100 breaths/min. The inspired O_2_ fraction was maintained at 0.21. VF was then induced through a guide wire advanced into the right ventricle. Using a 60 Hz current, a progressive increase from 2.7 mA to a maximum of 5 mA was then delivered to the right ventricular endocardium. The current was maintained for 3 min to prevent spontaneous defibrillation. Mechanical ventilation was discontinued after the onset of VF. PC, together with mechanical ventilation (tidal volume 0.60 mL/100 g body weight, 100 breaths/min, inspired O_2_ fraction 1.0), was initiated after 6 min of untreated VF, using a pneumatically driven mechanical chest compressor. PC was maintained at a rate of 200 breaths/min and was synchronized to provide a compression-to-ventilation ratio of 2 : 1, with equal compression and relaxation periods, for a duration of 8 min. The depth of PC was initially adjusted to decrease the anteroposterior diameter of the chest by 25% and to maintain coronary perfusion pressure (CPP) at 22 ± 2 mmHg. Resuscitation was attempted with up to three 2 J countershocks. ROSC is defined as the return of spontaneous circulation with a mean aortic pressure (MAP) > 50 mmHg for 5 min. If ROSC was not achieved, a 30-second interval of PC was performed before attempting a subsequent sequence of up to three countershocks. The procedure was repeated for a maximum of 3 cycles. If ROSC was not achieved, the resuscitation maneuvers were ended. After ROSC, mechanical ventilation was continued with 100% inspired oxygen for 1 h, 50% for 1 h, and 21% thereafter. During this time, the animals uniformly recovered from anesthesia. The rat hearts from the ROSC 6 h subgroups were rapidly excised and stored at −20°C freezer for further study. Six hours after ROSC, all catheters, including the endotracheal tube, were removed from the ROSC 24 h subgroups. Butorphanol (0.4 mg/kg) was injected intramuscularly if discomfort was identified. The rats are returned to cages equipped with heat lamps to maintain the cage temperatures between 24°C and 26°C. All surviving animals were euthanized by intraperitoneal injections of pentobarbital (150 mg/kg) after a 24 h observation period. An autopsy was performed on each animal to inspect for gross abnormalities, including evidence of traumatic injuries consequent to cannulation, airway management, or precordial compressions.

### 2.3. Hemodynamic Parameter Measurements

AP, RAP, EKG, and ETCO_2_ values were recorded continuously on a personal computer-based data-acquisition system supported by Common Ocean Data Access System hardware and software (DataQ, Akron, Ohio). CPP was calculated digitally based on the differences between time-coincident diastolic aortic pressure and RAP values measured at the end of each minute of PC. Acute ECG changes after CPR and defibrillation shocks were recorded by continuous ECG monitoring. At BL and postresuscitation (PR) 1, 4, and 6 h, myocardial function values, including ejection fraction (EF), cardiac output (CO), and myocardial performance index (MPI), were measured by echocardiography. The MPI, which combines time intervals related to systolic and diastolic functions and reflects global cardiac function, was also calculated using the formula (*a*-*b*)/*b*, where *a* was the mitral closure-to-opening interval (time interval from cessation to onset of mitral inflow) and *b* was the aortic flow ejection time, obtained at the LV outflow tract.

### 2.4. Determination of IL-6, TNF-*α*, and NT-proBNP Levels in Serum

Arterial blood samples (1 ml) were withdrawn at BL and PR 6 h to determine serum concentrations of IL-6, TNF-*α*, and NT-proBNP, which were measured using commercial enzyme-linked immunosorbent assay (ELISA) kits (Wuhan CUSABIO Biotech Industry Co., Ltd. China).

### 2.5. Heart Histopathologic Analysis and 24-Hour Survival Rate

Heart tissues from random samples were fixed immediately in 4% paraformaldehyde and embedded in paraffin following the end of each experiment. Tissue blocks were stained with hematoxylin and eosin (H&E) and evaluated under light microscopy. Myocardial injuries were identified by perivascular edema, neutrophil infiltration, and myofibril derangement [22]. Histopathologic evaluations were analyzed by three independent observers who were blinded to the experiment. The 24 h survival rate after CPR was monitored and compared between the CPR + rhBNP and CPR + placebo groups for all resuscitated animals.

### 2.6. Western Blotting Analysis

Heart tissue was obtained according to the published protocol [23]. After a 6 h monitoring period, 12 rats were sacrificed under anesthesia, following the animal care protocol. Ventricular muscle tissue protein homogenates were separated via 10% sodium dodecyl sulfate-polyacrylamide gel electrophoresis and transferred onto a polyvinylidene fluoride (PVDF) membrane, which was then blocked in 5% skimmed milk powder for 1 h. Primary antibodies directed against IL-6 (1 : 1000), TNF-*α* (1 : 600), TLR4 (1 : 400), p65 (1 : 1,000), and phosphor-p65 (1 : 1,000) (Santa Cruz, Dallas, Texas, USA, and Cell Signaling, Danvers, MA, USA) were added and incubated overnight at 4°C. After washing in Tris-buffered saline containing Tween-20 (TBST), membranes were then incubated with secondary antibody for 1 h at room temperature, and proteins were detected with an enhanced chemiluminescence kit. The product ratio and the internal standard grayscale values were calculated using ImageJ 1.38 × software (National Institutes of Health, Bethesda, MD, USA).

### 2.7. Statistical Analysis

The experimental data were analyzed by Statistical Product and Service Solutions 22.0 (Chicago, Illinois). Measurements are reported as the mean ± standard deviation (SD). Comparisons between time-based measurements within each group were performed using a repeated-measures analysis of variance (ANOVA). Survival analysis was performed using the Kaplan–Meier method. A value of *p* < 0.05 was regarded as being statistically significant.

## 3. Results

### 3.1. Characteristics of Rats at BL, before Inducing VF

Twenty-one rats were used in our study, and five rats were excluded due to technical or experimental instrument failures during animal preparation and resuscitation. Body weights, BL measurements of hemodynamics and myocardial function, and serum levels of inflammatory cytokines and myocardial tissue injury biomarkers (IL-6, TNF-*α*, and NT-proBNP) were not significantly different between the two groups (*p* > 0.05) (Table 1).

### 3.2. Myocardial Tissue Injury

**Figure 1:**
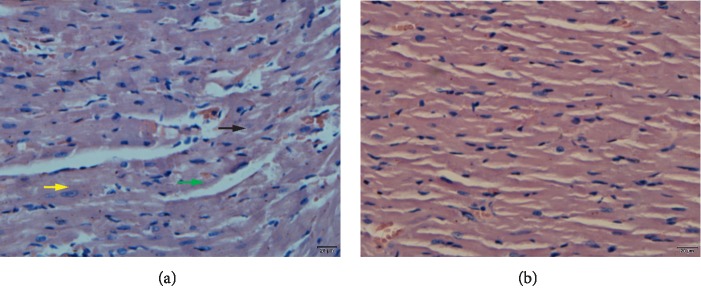
Representative myocardial tissue morphological injuries in the CPR + placebo (a) and CPR + rhBNP groups (b). Myocardial injury in the rhBNP group was reduced compared with the placebo group, as characterized by perivascular edema (green arrows), myofibril derangement (black arrows), and neutrophil adhesion (yellow arrows) to the vascular endothelium.

**Figure 2:**
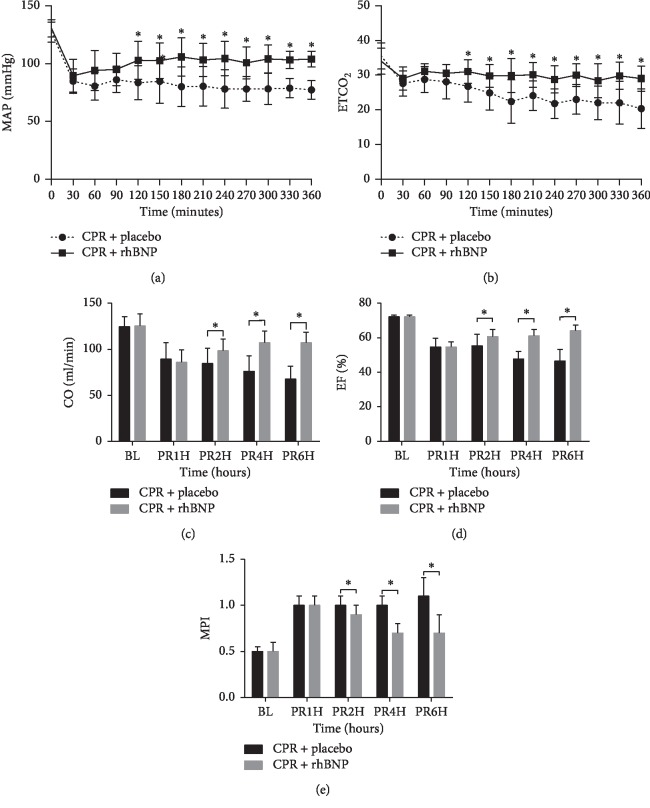
Comparison of MAP (mean aortic pressure), ETCO_2_, CO (cardiac output), EF (ejection fraction), and MPI (myocardial performance index) values between the two groups 6 h postresuscitation (PR). ^*∗*^*p* < 0.05, CPR + placebo group vs. CPR + rhBNP group. BL: baseline.

All resuscitated rats in both groups had obvious myocardial tissue injuries compared with baseline measurements. The severity of the myocardial tissue injuries in the CPR + rhBNP group was significantly reduced compared with the CPR + placebo group, which was characterized by reduced perivascular edema, myofibril derangement, and neutrophil infiltration (Figure 1). All animals in both groups were resuscitated with significant PRMD, as measured by MAP, ETCO_2_, EF, CO, and MPI, when compared with the baseline parameters (*p* < 0.05). The administration of rhBNP significantly improved the severity of PRMD (*p* < 0.05) (Figure 2).

### 3.3. 24-Hour Survival Rate of Rats

**Figure 3:**
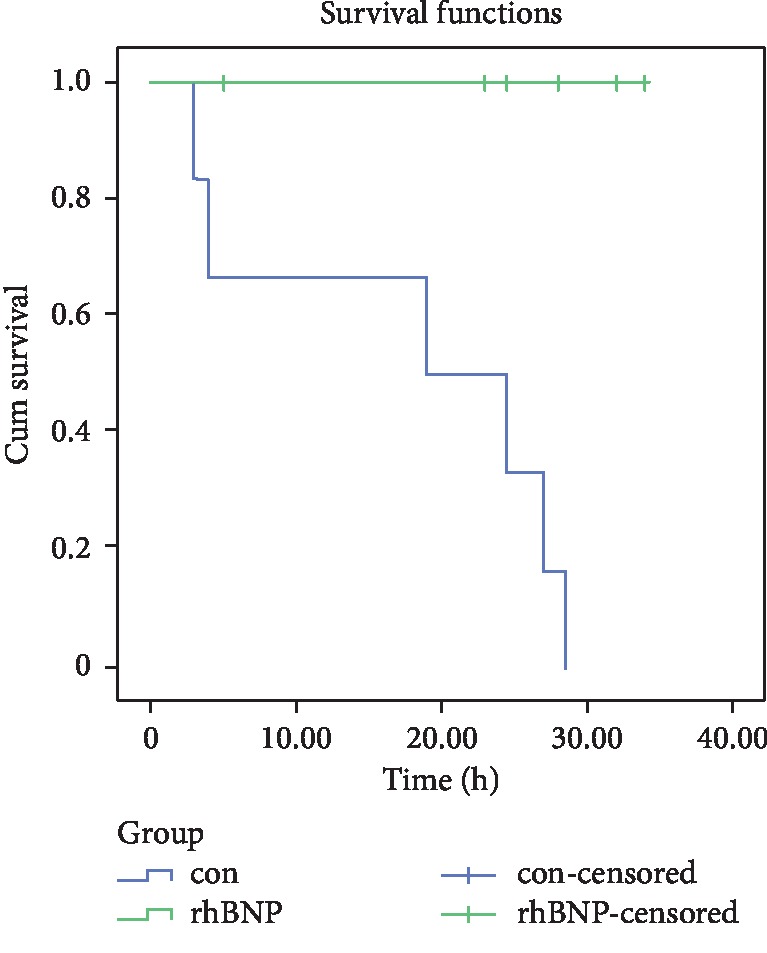
Kaplan–Meier survival curves for the two groups. A log-rank test indicated that the 24 h survival rate was significantly different between the CPR + placebo and CPR + rhBNP groups.

A log-rank test showed a significant difference between the 24 h survival rate curves of the CPR + placebo and CPR + rhBNP groups (*p* < 0.05) (Figure 3).

### 3.4. Serum Levels of IL-6, TNF-*α*, and NT-ProBNP and Expressions of IL-6 and TNF-*α* in Myocardial Tissue

**Figure 4:**
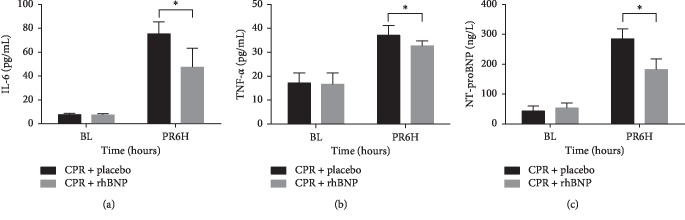
Comparison of IL-6, TNF-*α*, and NT-proBNP levels between the two groups 6 h postresuscitation (PR). ^*∗*^*p* < 0.05, CPR + placebo group vs. CPR + rhBNP group. BL: baseline.

**Figure 5:**
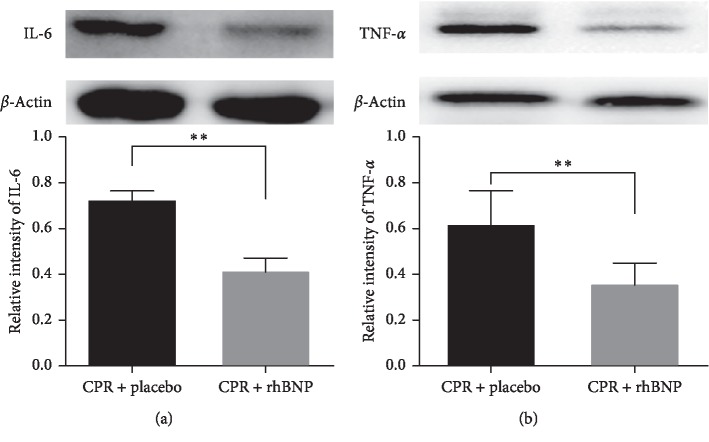
Comparison of the IL-6 and TNF-*α* expression levels in myocardial tissue between the two groups 6 h postresuscitation (PR). ^*∗*^*p* < 0.05, CPR + placebo group vs. CPR + rhBNP group. BL: baseline.

The serum levels of IL-6, TNF-*α*, and NT-proBNP significantly increased after ROSC in both groups when compared with baseline levels. The administration of rhBNP significantly decreased the serum levels of IL-6, TNF-*α*, and NT-proBNP in the CPR + rhBNP group when compared with those in the CPR + placebo group (*p* < 0.05) (Figure 4). Expressions of IL-6 and TNF-*α* in myocardial tissue were significantly decreased in the CPR + rhBNP group (*p* < 0.05) (Figure 5).

### 3.5. TLR4/NF-*κ*B/p-p65/p65 Signaling Pathway

**Figure 6:**
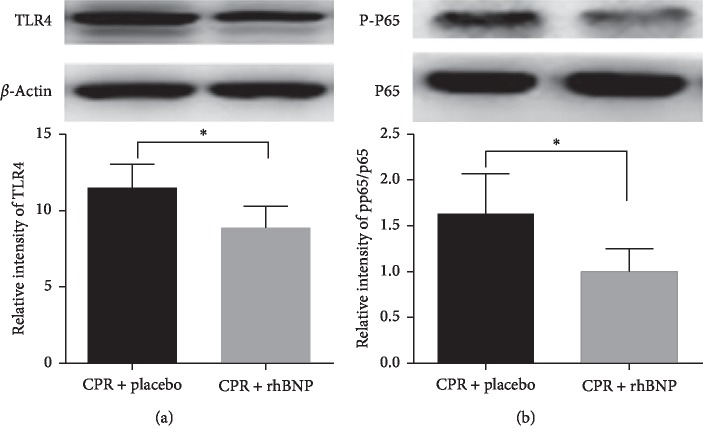
Comparison of the TLR4 and p-p65/p65 expression levels between the two groups 6 h postresuscitation (PR). ^*∗*^*p* < 0.05, CPR + placebo group vs. CPR + rhBNP group. BL: baseline.

The protein expression levels of p65, p-p65, and TLR4 were significantly decreased after PR 6 h in the CPR + rhBNP group when compared with those in the CPR + placebo group (*p* < 0.05) (Figure 6).

## 4. Discussion

This study demonstrated that the administration of rhBNP significantly decreased the severity of PRMD and myocardial tissue injuries and improved hemodynamic stability and the 24 h survival rate in a rat CA model. The protective effects of rhBNP on PRMD are likely to be associated with the inhibition of the TLR4/NF-*κ*B signaling pathway, resulting in the reduced synthesis and secretion of inflammatory cytokines (IL-6 and TNF-*α*) in myocardial tissue and serum. The current treatment protocol for CA has become increasingly intricate, with an ever-growing array of therapeutic methods being utilized to prevent sudden death [24]. PRMD has been identified as the leading cause of death during the 72 h PR period [25]. Therefore, the study of PRMD and the identification of drugs to treat PRMD have attracted much attention. Our previous research demonstrated that the serum concentration of NT-proBNP was significantly positively correlated with the severity of myocardial dysfunction and mortality [6]. Based on basic and clinical studies, the protective effects and safety of rhBNP administration during acute myocardial infarctions, acute heart failure, and the perioperative period of cardiac surgery have gradually been recognized, which provides the theoretical basis for our research [7, 26, 27]. In the present study, the administration of rhBNP significantly alleviated the severity of PRMD, myocardial inflammatory response, and myocardial tissue injuries, as reflected by improvements in MAP, ETCO_2_, EF, CO, and MPI values, myocardial inflammatory factors (IL-6 and TNF-*α*), serum levels of NT-proBNP, and myocardial tissue morphological injuries. The results of rhBNP administration on PRMD and the 24 h survival rate in a rat CA model suggest that this drug may have further clinical applications and research value for CA patients.

Another focus of this study is the molecular mechanism underlying the myocardial protection provided by rhBNP in a rat CA model. The excessive release of inflammatory cytokines after ROSC has been observed, which may be triggered by pathophysiological abnormities during PRS [28]. Many studies have demonstrated that inflammation and inflammatory factors play important roles in the pathophysiological mechanism underlying myocardial ischemia-reperfusion injuries, and increased expression levels of IL-1*β*, IL-6, IL-8, TNF-*α*, interferon-gamma, and intercellular adhesion molecule-1 have been observed in myocardial tissue [29]. Our research showed that CA and resuscitation significantly increased the release of IL-6 and TNF-*α* in serum and myocardial tissue, and the administration of rhBNP significantly decreased the serum levels and myocardial tissue of IL-6 and TNF-*α*. These results suggest that the protective mechanism of rhBNP may be related to its anti-inflammatory mechanism. Although no related studies examining myocardial protection and the mechanism of rhBNP action have been reported, some studies have shown that rhBNP plays an anti-inflammatory role (based on decreased expression levels of IL-6 and TNF-*α*) in lung, kidney, and intestinal tissues from animal models of liposaccharide- (LPS-) induced acute injuries [26, 30, 31]. These results suggest that rhBNP may have a protective effect against multiple organ dysfunction induced by ischemia-reperfusion injuries and sepsis, which requires further research.

TLR4 acts as a pattern-recognition receptor, which contributes to the inflammatory reaction induced by I/R injuries [32]. TLR4-deficient mice experienced reduced myocardial infarct sizes after myocardial I/R injuries that were mediated by the reduced activity of inflammatory signaling pathways, such as the accumulation of polymorphonuclear neutrophils and the activation of oxidative stress [33]. NF-*κ*B is an important proinflammatory transcription factor [34]. The activation of TLR4 has been associated with the expression of proinflammatory cytokines (IL-1*β*, IL-6, and TNF-*α*) and the activation of the NF-*κ*B signaling pathway in several cell types [35–37]. The histopathologic analysis of the heart showed that rats in the rhBNP group had reduced accumulations of polymorphonuclear neutrophils. The expression levels of TLR4 and NF-*κ*B were decreased in the rhBNP group when compared with the placebo group. These results suggest that the protective action of rhBNP against I/R injury is associated with the downregulation of TLR4/NF-*κ*B and the inhibition of the inflammatory response. Li et al. reported that rhBNP can reduce the expression levels of IL-6 and TNF-*α* in LPS-activated RAW 264.7 macrophage cells and peripheral blood mononuclear cells by inhibiting the NF-*κ*B and MAPK pathways [17]. Therefore, the anti-inflammatory effects and mechanisms of rhBNP have broad research and clinical application prospects.

There were some limitations of our study. First, this study was performed in animals without any underlying diseases related to cardiac arrest. Second, rhBNP was administered by intravenous infusion for 6 h after ROSC, as a representation of the early stage of PRMD. Finally, we did not examine different doses of rhBNP in this study. Based on our findings, we plan to conduct further studies examining the effects of different rhBNP doses.

In summary, the administration of rhBNP decreased the severity of PRMD and myocardial tissue injuries, with improvements in the 24 h survival rate in a rat CPR model. The protective effect of rhBNP was likely caused by the suppression of the TLR4/NF-*κ*B pathway and the regulation of inflammatory mediator secretion. This finding has important implications for the clinical improvement of PRMD and myocardial tissue injuries induced by CA and resuscitation.

## Figures and Tables

**Table 1:** Baseline characteristics.

Group	CPR + placebo	CPR + rhBNP	Sham
Body weight (g)	484.7 ± 33.3	486.3 ± 19.6	494.3 ± 12.1
Heart rate (bpm)	413.6 + 21.8	408.8 + 23.0	404.2 + 19.8
MAP (mmHg)	127.3 ± 8.8	130.5 ± 7.6	130.9 ± 7.3
ETCO_2_ (mmHg)	35.5 ± 3.8	34.0 ± 3.8	35.5 ± 2.9
Temperature (°C)	36.8 ± 0.2	37.0 ± 0.3	36.7 ± 0.3
CO (ml/min)	124.4 ± 10.8	125.3 ± 13.3	123.8 ± 6.2
EF (%)	71.9 ± 1.4	72.2 ± 1.2	72.3 ± 1.1
MPI	0.5 ± 0.05	0.5 ± 0.0	0.5 ± 0.0
Lactate (mmol/L)	0.8 ± 0.1	0.7 ± 0.2	0.7 ± 0.1
NT-proBNP (ng/L)	42.9 ± 17.2	52.8 ± 17.4	52.8 ± 16.3
IL-6 (pg/mL)	7.8 ± 1.1	7.5 ± 1.0	7.0 ± 2.7

MAP: mean aortic pressure; CO: cardiac output; EF: ejection fraction; MPI: myocardial performance index. Values are presented as the mean ± SD.

## Data Availability

The data used to support the findings of this study are included within the article.
